# Occupational Therapy Interventions to Improve the Quality of Life of Older Adults with Dementia Living in Nursing Homes: A Systematic Review

**DOI:** 10.3390/healthcare12090896

**Published:** 2024-04-25

**Authors:** Cristian Uceda-Portillo, Sandra Aranda-Valero, Pedro Moruno-Miralles

**Affiliations:** 1 Department of Psychology, National University of Distance Education (UNED), Talavera de la Reina, 45600 Toledo, Spain; 2 Department of Nursing and Physiotherapy, University of Salamanca, 37008 Salamanca, Spain; idu052535@usal.es; 3 Department of Nursing, Physiotherapy and Occupational Therapy, University of Castilla-La Mancha, Talavera de la Reina, 45600 Toledo, Spain; pedro.moruno@uclm.es

**Keywords:** occupational therapy, quality of life, older adults, dementia, nursing homes

## Abstract

The increase in older adults with dementia presents challenges in promoting research to improve the quality of life of this population. The objective of this study was to assess the scientific evidence on the effectiveness of occupational therapy interventions in improving the quality of life of older adults over 65 years old with dementia living in nursing homes. The databases used were PubMed, Web of Science, OTSeeker, clinicaltrials.gov, Dialnet, Scopus, Cochrane, and SciELO between 2013 and 2023. The studies were selected and evaluated according to the Cochrane guidelines. The review was carried out following the PRISMA 2020 Statement. Sixteen articles met the inclusion criteria and were categorized into four groups according to the focus of the intervention: “meaningful activities/occupations”, “physical, cognitive and sensory functioning”, “performance areas”, and “physical and social environment and staff training”. The strength of evidence was moderate, and the risk of bias was low. The findings revealed that occupational therapy interventions based on participation in recreational activities, reminiscence, performance-based activities and the physical and social environment, and specialized staff training, could improve the perceived quality of life of older adults with dementia living in nursing homes.

## 1. Introduction

Dementia is a major neurocognitive disorder characterized by significant cognitive decline compared to the previous level of performance in one or more cognitive domains (complex attention, executive function, learning and memory, language, perceptual motor function, or social cognition) which interferes with the individual’s autonomy in daily activities [1].

Currently, more than 55 million older adults over 65 years old (8.1% of women and 5.4% of men) have dementia worldwide. This is expected to rise to 78 million by 2030 and 139 million by 2050 [2]. In addition, the prevalence of dementia among older adults living in nursing homes is highly variable, ranging from 16.1% to 85.2%, depending on factors such as the country, the method and timing used to conduct the research, and the degree of aging [3]. Therefore, addressing dementia has become a public health priority and a major socio-health problem, as its scope, size, and socio-economic impact present society with the challenge of promoting study and research to improve the well-being and quality of life (QoL) of this population group [4].

QoL is a multidimensional concept that has evolved throughout history and currently has multiple interpretations. Thus, it is difficult to find a single definition [5].

An integrative definition of the concept of QoL has been proposed by Fernández-Ballesteros [6], in which she proposes two classifications: the first one separates the socio-environmental factors (social support, financial conditions, health and social services, environmental quality, and cultural factors) and the personal factors (social relations, life satisfaction, participation in meaningful activities, health, and functional skills); and the second distinguishes between objective elements (physical environment, availability of social health services, objective health, social networks, and cultural factors) and subjective elements (health, social satisfaction, cultural needs, context evaluation, and functional skills). Taking all these factors into account, the concept of QoL assesses different dimensions of the person’s life, through a comprehensive and complex approach.

Interventions from an occupational therapy (OT) perspective could be offered to older adults with dementia living in nursing homes, where they are provided with temporary or permanent accommodation and appropriate programs to improve their QoL and personal autonomy [7]. These interventions focus on and consider variables such as mental state, physical functioning, characteristics of the residential environment, health promotion, activities of daily living (ADLs), instrumental activities of daily living (IADLs), health management, education, leisure, and social participation [8]. From this perspective, OT interventions represent a vehicle for the promotion and maintenance of autonomy, health, and QoL of older adults with dementia [9].

However, previous literature reviews could not identify the existence of current systematic reviews summarizing the scientific evidence on the effectiveness of OT interventions in this particular population and setting. In the most recent systematic reviews on this topic, the population consisted of either healthy older adults [10] or patients with dementia but not as the primary diagnosis [11,12], or the study setting was not a nursing home [13,14].

Therefore, this study aims to systematically identify, evaluate, and summarize the scientific evidence on OT interventions to improve the QoL of older adults aged 65 and over with dementia living in nursing homes. The research question that guided the review was: What is the quality of the scientific evidence on the effectiveness of OT interventions to improve the QoL of older adults over 65 years old with dementia living in nursing homes?

## 2. Method

This systematic review was conducted following the Cochrane Collaboration methodology [15] and was reported according to the Preferred Reporting Items for Systematic Reviews and Meta-Analyses guidelines (PRISMA 2020) [16].

### 2.1. Search and Screening Strategy

The initial search process was carried out by the authors of this work in collaboration with a medical librarian experienced in conducting systematic reviews.

A formal literature search was conducted (10 July 2023 and 20 July 2023) in the following selected databases: PubMed, Web of Science (WOS), OTSeeker, clinicaltrials.gov, Dialnet, Scopus, Cochrane, and SciELO, using MeSH terms and keywords: “dementia”, “occupational therapy”, “quality of life”, “aged”, and “nursing home”. Studies conducted from 2013 to 2023 in any language and country were included. The latest information has therefore been compiled as comprehensively as possible. We aimed to avoid any bias that might affect the information collected.

The search string used in the databases was:

− PubMed: (((dementia) AND (quality of life)) AND (aged)) AND (occupational therapy) AND (nursing home) → 60 results obtained.
− WOS: ((((ALL=(dementia)) AND ALL=(quality of life)) AND ALL=(aged))) AND ALL=(occupational therapy) → 264 results obtained.
− OTSeeker: [Any Field] like ‘dementia’ AND [Any Field] like ‘quality of life’ AND [Any Field] like ‘occupational therapy’ → 86 results obtained.
− clinicaltrials.gov: (dementia AND occupational therapy) → 51 results obtained.
− Dialnet: (occupational therapy, quality of life, dementia) → 67 results obtained.
− Scopus: (dementia AND quality AND of AND life AND occupational AND therapy AND nursing AND home) → 194 results obtained.
− Cochrane: (dementia AND quality of life AND occupational therapy AND nursing home) → 223 results obtained.
− SciELO: (dementia AND quality of life AND occupational therapy) → 22 results obtained.

### 2.2. Inclusion and Exclusion Criteria

The following eligibility criteria were established.

→ Inclusion criteria:
  − Studies involving OT interventions in nursing homes.
  − Studies in older adults over 65 years old of both genders with a formal diagnosis of dementia of any type and stage [1].
  − Studies with a level of evidence 1a–1b to 2a–2b.
  − Studies that include the MeSH terms in the keyword list.
→ Exclusion criteria:
  − Studies with a primary focus on intervention other than OT and nursing homes. Older adults living at home or with their family, in the community, in hospitals, and in palliative care facilities.
  − Studies that include healthy older adults or older adults with dementia but not as the primary diagnosis.
  − Studies that do not contain any of the keywords.

Level 1a (systematic reviews of homogeneous randomized controlled trials [RCTs] with or without meta-analysis), level 1b (properly designed individual RCTs), level 2a (systematic review of cohort studies), and level 2b (individual prospective cohort studies, low-quality RCTs, ecological studies, two-group non-randomized studies) studies were included. Level 3a (systematic review of case-control studies), level 3b (individual retrospective case-control studies, non-randomized one-group pretest-post-test studies, and cohort studies), level 4 (case-series and poor-quality cohort and case-control studies), and level 5 (expert opinion without explicit critical appraisal: protocols, dissertations and theses, and editorials) studies were excluded [17].

### 2.3. Data Extraction and Analysis

Relevant information was collected from each included study and entered into a data collection form based on Cochrane recommendations [15], using Microsoft Excel^®^, version 16.16.21 software. Data collection was carried out independently by the researchers C.U. and S.A. Subsequently, the results of each investigator were compared until a consensus was reached. In addition, the entire data collection process was independently supervised by a third researcher (P.M.). Information on the following variables was collected: author/year, level of evidence, study design, risk of bias, participants, inclusion criteria, study setting, intervention and control group, outcome measures, and results. Finally, with the purpose of improving the comprehension, readability, and organization of the information presented on the OT intervention programs analyzed, the researchers C.U. and S.A. consensually grouped the studies according to the main objective or focus of intervention. In addition, a third researcher (P.M.) independently supervised the entire categorization process. Finally, the studies were categorized into four groups: (a) “meaningful activities/occupations”, (b) “physical, cognitive and sensory functioning”, (c) “performance areas”, and (d) “physical and social environment and staff training” (see Table 1, Table 2, Table 3 and Table 4).

### 2.4. Risk of Bias

The risk of bias (low, moderate, or high) in each study included in the systematic review was assessed according to the Cochrane risk of bias assessment guidelines [33]. The guidelines for carrying out a Cochrane risk of bias assessment cover five domains of bias: selection bias, performance bias, detection bias, attrition bias, and information bias. Within each domain, the assessment is performed for one or more items, which may cover different aspects of the domain. To determine the overall risk of bias, a risk of bias category must first be assigned for each item. The categories for risk of bias are as follows: low risk of bias (+), unclear risk of bias (?), and high risk of bias (−). The total number of minuses (−) is then summed. Finally, the overall risk of bias in each study is classified as low (L) (0–3 minuses), moderate (M) (4–6 minuses), or high (H) (7–9 minuses). The risk of bias in the systematic reviews included was assessed using the AMSTAR 2 guidelines [34]. The AMSTAR 2 guidelines consist of 12 assessment items, each of which relates to specific aspects of the method used to conduct the systematic review. To determine the overall risk of bias, each item must first be assessed by determining whether the systematic review meets that criterion by assigning a yes (+), no (−), not sure (?), or not applicable (NA). The total number of minuses (−) is then summed. Finally, the overall risk of bias in each study is classified as low (L) (0–3 minuses), moderate (M) (4–6 minuses), or high (H) (7–9 minuses). Two researchers (C.U. and S.A.) independently assessed the risk of bias. The results were then compared collaboratively to reach a consensus. Next, the results were independently reviewed by a third researcher (P.M.). Table 5 and Table 6 show the risk of bias assessment of the included studies.

### 2.5. Overall Strength of Evidence

The strength of the evidence was assessed based on the guidelines developed by the U.S. Preventive Services Task Force [35]. In short, for each topic, the levels of strength of evidence are high strength of evidence, which consists of two or more well-designed RCTs whose conclusions are unlikely to be affected by the results of future studies; moderate strength of evidence, which consists of at least one high-quality RCT or multiple moderate quality studies; and the low strength of evidence, which involves a limited number of incomplete and low-quality studies.

## 3. Results

**Figure 1:**
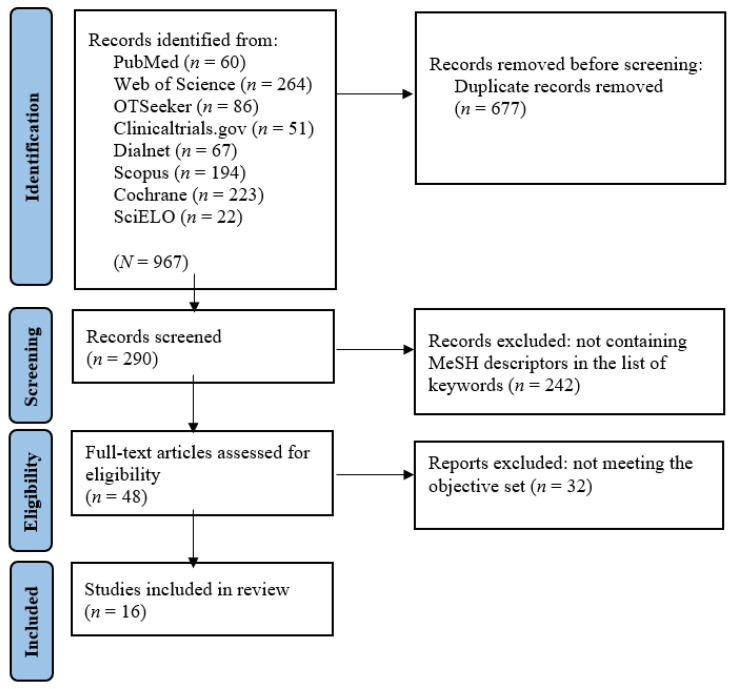
PRISMA flow diagram. *Note.* Figure format adapted from Moher et al. [36].

The literature search identified 967 studies, of which 48 were subjected to a full-text review. Sixteen studies met the eligibility criteria and were included in the analysis (see Figure 1).

Sixteen studies involved OT interventions to improve the QoL of older adults with dementia living in nursing homes. Three level 1a studies were identified [9,18,30]. Twelve level 1b studies were identified [19,20,21,22,24,25,26,27,28,29,31,32]. A single level 2b study was identified [23]. Fifteen studies showed a low risk of bias, and one study met the criteria for a moderate risk of bias (see Table 1, Table 2, Table 3, Table 4, Table 5 and Table 6).

The sixteen studies were categorized into four groups according to the primary objective or focus of intervention: (a) “meaningful activities/occupations”, (b) “physical, cognitive and sensory functioning”, (c) “performance areas”, and (d) “physical and social environment and staff training”. For group (a) “meaningful activities/occupations”, four studies (25.0%) were identified [18,19,20,21]. For group (b) “physical, cognitive and sensory functioning”, six studies (37.5%) were identified, which were divided into two subgroups: (b1) physical activity [22,23], and (b2) cognitive and sensory functioning [24,25,26,27]. Regarding group (c) “performance areas”, three studies (18.7%) were included [28,29,30]. Regarding group (d) “physical and social environment and staff training”, three studies (18.7%) were identified [9,31,32]. The characteristics of each study and their outcome measures are described in Table 1, Table 2, Table 3 and Table 4.

**(a)**  **Meaningful activities/occupations**

Meaningful activities/occupations were the primary intervention in four of the included studies [18,19,20,21].

Mansbach et al. [19] (level 1b evidence) conducted an intervention program called “MemPics”, designed to promote meaningful activities and improve QoL for older adults with dementia by engaging them in verbal activities (e.g., fun and stimulating conversations, prompting questions for further conversation) and cognitive stimulation (e.g., reminiscence therapy). The results showed increased engagement in meaningful activities (Engagement in Meaningful Activities Survey [EMAS]) and improved the QoL of the intervention group (assessed with the MemPics Facility Staff Survey).

Livingston et al. [20] (level 1b evidence) developed an intervention program called “MARQUE”, designed to promote agitation management and improve QoL in older adults with dementia through their participation in six sessions (psycho-education on dementia, staff experiences in agitation management, stress reduction techniques, communication, a “Call to Mind” board game to discover participants’ interests, and incorporation of meaningful activities into the daily care of older adults). After the 8-month follow-up, no significant differences were observed between the groups for the level of agitation and QoL (assessed with the Dementia Quality of Life [DEMQOL] and the EuroQol-5D-5L [EQ-5D-5L]).

Sultan Ibrahim et al. [21] (level 1b evidence) conducted a program called “Occupation-based intervention”, consisting of cognitive activities (e.g., image recognition and categorization, memory, sensory recognition) as well as meaningful occupational activities (e.g., personal hygiene, cooking, money management, shopping, leisure, and recreational activities). The results showed a significant improvement in cognitive function (evaluated with the Lowenstein Occupational Therapy Cognitive Assessment-Geriatric [LOTCA-G]), social relations (measured with the Friendship Scale [FS]), and QoL (assessed with the Brief Version of World Health Organization-Quality of Life [WHOQOL-BREF]) of the participants.

The results of the above studies are consistent with those of the systematic review with meta-analysis conducted by Travers et al. [18] (level 1a evidence) to determine the effectiveness of the use of meaningful activities (individualized recreational activities, reminiscence therapy, music therapy, multi-sensory stimulation, staff training to provide individual care, animal-assisted therapy, and social interaction) in addressing behavioral and psychological symptoms (agitation, aggression, depression, wandering, and apathy), and improving the QoL of older adults with dementia. The results revealed beneficial effects as a result of the promotion of individualized recreational activities, reminiscence therapy, and music therapy on the reduction of agitation, depression, and anxiety, as well as an improvement in cognitive functioning and QoL of the residents.

**(b)**  **Physical, cognitive, and sensory functioning**

This section presents the studies grouped into sub-themes, according to the main findings of each study.

(*b*1)  *Physical activity*

Physical activity was the primary intervention in two studies [22,23].

Galik et al. [22] (level 1b evidence) conducted a function-focused care intervention program for older adults with dementia (FFC-CI). The intervention included four components: (I) assessment of the physical environment of the nursing home in order to identify architectural barriers when implementing the intervention; (II) training program on FFC-CI for the nursing home staff; (III) development of function-focused care goals (active participation of residents in self-care, household, mobility, physical activity, and dance); and (IV) continuous training and motivation of staff to involve residents in activities that promote their activity and functioning. The results showed significant improvements in the amount and intensity of physical activity (measured with ActiGraph) and physical function (assessed with the Tinetti Scale and Barthel Index [BI]) of older adults, as well as a decrease in the number of falls in the intervention group, resulting in an improvement in their QoL.

Chu et al. [23] (level 2b evidence) carried out a quasi-experimental study consisting of the implementation of a program called “Multifaceted Walking Intervention”, which included low-intensity physical activity (walking session) and an individualized care plan (communication, social interaction, behavior, personality, values, and preferences of the resident). After a four-month intervention, the results showed significant improvements in the functional mobility (Timed Up and Go Test [TUG] and 2-Minute Walk Test [2MWT]), ADLs (Functional Independence Measure [FIM]), and QoL (Alzheimer’s Disease-Related Quality of Life [ADRQOL] Scale) of the participants.

(*b*2)  *Cognitive and sensory activities*

The primary intervention focused on cognitive and sensory functioning in four studies [24,25,26,27].

Maseda et al. [24] (level 1b evidence) assessed the effect of multisensory stimulation on the behavior, mood, and cognitive and functional levels of residents with dementia. To this end, a “Snoezelen” room with different elements for the stimulation of the senses (e.g., fiber optic cables, water columns, a vibrating waterbed, screen projectors, different music and sounds, aromatherapy, and different textures) and individualized activities (e.g., playing cards, taking questionnaires, and looking at photographs) were used. The results showed significant improvements in the behavior (measured with the Cohen-Mansfield Agitation Inventory [CMAI]), cognitive level (evaluated with the Mini-Mental State Examination [MMSE]), and ADLs (assessed with the BI) of the intervention group.

Raglio et al. [25] (level 1b evidence) conducted a study to explore the effects of an intervention based on music therapy and individualized listening to music on QoL, behavior, and mood in older adults with dementia. Participants were randomly assigned to one of the following three interventions: (I) standard care, which consists of physical (e.g., motor rehabilitation), educational, and occupational activities (e.g., self-care, reading the newspaper, playing cards), with no musical exposure; (II) music therapy and standard care, based on the use of instruments, singing, rhythm, and music production; and (III) individualized listening to music and standard care, focused on listening to personalized music on a one-to-one basis. The findings revealed significant improvements in QoL (measured with the Cornell-Brown Scale for Quality of Life in Dementia [CBS-QoL]), behavior (assessed with the Neuropsychiatric Inventory [NPI]), and mood (measured with the Cornell Scale for Depression in Dementia [CSDD]) for all groups, regardless of the intervention received.

Lök et al. [26] (level 1b evidence) explored the effect of reminiscence therapy on the QoL, cognitive function, and mood of participants. The sessions included recalling memories of childhood experiences, festivals, memorable places visited, favorite foods and music, major historical events, and achievements, using different materials such as photographs, household items, objects from the past, old music, and food. The results indicated a significant improvement in the cognitive function (measured with the MMSE), depressive symptoms (evaluated with the CSDD), and QoL (assessed with the QOL-AD) of older adults with dementia.

Kim [27] (level 1b evidence) explored the effectiveness of a reminiscence-based program on cognitive function, mood, and QoL of residents with dementia. The sessions included physical, musical, artistic, and horticultural activities, and IADLs. Each activity was divided by content according to childhood, adulthood, and late adulthood memories. The results showed a significant improvement in the cognitive function (measured with the Korean-Mini-Mental State Examination [K-MMSE]), depression (assessed with the Short-Form Geriatric Depression Scale-K [SGDS-K]), and QoL (measured with the QOL-AD Scale) of participants.

**(c)**  **Performance areas**

In three studies, performance-based activities were the primary intervention [28,29,30].

Kumar et al. [28] (level 1b evidence) explored the effects of an OT program to improve the QoL of older adults with dementia, through their participation in ADLs (care of hair, skin, nails and teeth, general cleanliness, dressing), IADLs (bed making, money counting), physical activity (exercises aimed at maintaining strength, mobility, and circulation), cognitive activities (reading aloud, dual-task activity, solving puzzles), recreational activities (watching TV, board games, quizzes, storytelling, singing), relaxation exercises, and pharmacological treatment. The results showed an improvement in the QoL (measured with the WHOQOL-BREF) of the participants.

Murai and Yamaguchi [29] (level 1b evidence) assessed the effects of a cooking program based on the principles of brain-activating rehabilitation on the QoL, executive function, behavior, mood, and ADLs of the participants. The program consisted of cooking 12 homemade Japanese-style dishes (e.g., miso soup with tofu and seaweed, *udon* noodles), in which different activities, such as knife cutting, boiling, grilling, and seasoning, were carried out. The results showed significant improvements in the executive function (measured with the Yamaguchi Kanji-Symbol Substitution Test [YKSST]) and behavior (assessed with the Dementia Behavior Disturbance [DBD] Scale) of the participants.

The results of the above studies are consistent with those of the systematic review with a meta-analysis conducted by Möhler et al. [30] (level 1a evidence) to assess the effects of personally tailored activities (IADLs, such as household chores and meal preparation; artistic activities, such as painting and singing; work-related activities, such as gardening; and recreational activities, such as games) on the improvement of the psycho-social outcomes and QoL of older adults with dementia. This study concluded that offering personally tailored activities to people with dementia in long-term care could slightly improve challenging behavior.

**(d)**  **Physical and social environment and staff training**

Activities based on the physical environment, social environment, and staff training were the primary interventions in three studies [9,31,32].

Wenborn et al. [31] (level 1b evidence) developed an OT program that included an assessment of the physical environment of the nursing home, with recommendations on how to adapt and improve it to enable residents to be active. In addition, a training program for nursing home staff, consisting of group discussions, didactic teaching, and practical exercises was designed. This training program aimed, on the one hand, to improve the knowledge, attitudes, and skills of the staff to provide personally meaningful activities, and, on the other hand, to identify the interests and abilities of the residents to carry them out, in order to redesign and subsequently conduct new meaningful activities (self-care, domestic activities, music therapy, sensory stimulation, and physical exercise activities) adapted to each participant. At the quarterly follow-up, staff-rated QoL (measured with the Quality of Life in Alzheimer’s Disease—Patient and Caregiver Report [QOL-AD] Scale) was slightly lower in the intervention group.

Froggatt et al. [32] (level 1b evidence) conducted an intervention program called “Namaste Care”, focused on improving the physical environment, comfort, and sensory engagement of residents with dementia, in which personalized and structured care (creative activities, multisensory stimulation, social participation, and a training program for nursing home staff) was provided in a specific space (cozy and homely, with natural light, relaxing music, and aromatherapy). After a six-month intervention, the results revealed a significant improvement in the comfort (assessed with the Comfort Assessment in Dying-End of Life Care in Dementia [CAD-EOLD] Scale) of the participants.

The results of the above studies are consistent with those of the systematic review with meta-analysis conducted by Ojagbemi and Owolabi [9] (level 1a evidence), which aimed to explore the effects of OT interventions (compensatory and environmental modification activities; training for nursing home staff; relaxation exercises; sensorimotor activities, e.g., video viewing; recreational activities, e.g., playing musical instruments; cognitive activities, e.g., word games; and IADLs, e.g., caring for farm animals) on the QoL of older adults with dementia. This study concluded that OT interventions resulted in small improvements in the overall QoL of this population.

## 4. Discussion

This systematic review aimed to assess the scientific evidence on the effectiveness of OT interventions on improving the QoL of older adults over 65 years old with dementia living in nursing homes.

First, the intervention programs focused on meaningful activities and occupations and structured according to individual changes in activities based on the preferences and wishes of each participant, the type and stage of dementia, and the functional ability of the older adult with dementia to perform them [18,19,20], or specific programs of activities and occupations [21], show therapeutic effects on the behavioral and psychological symptoms of dementia, which in turn positively influence the perception of the QoL.

Individualized recreational activities/occupations such as music or painting show a high strength of evidence for the improvement of agitation, depression, anxiety, and mood. Also, reminiscence activities have positive effects on the cognitive functioning and QoL of residents [18].

Verbal and communication-enhancing activities [19] and meaningful occupational activities [21] show a moderate strength of evidence for the improvement of social relationships, cognitive function, and QoL. However, no improvement in agitation in older adults with dementia is observed with psycho-education and stress reduction activities [20].

Overall, these results are consistent with those of Testad et al. [37], which support the value of personalized enjoyable activities, with and without social interaction, for the treatment of dementia symptoms such as depression, anxiety, and challenging behavior. These interventions require the design of tailored activities to meet the individual characteristics of each participant. Therefore, OT professionals play a key role in selecting activities that are adapted to the needs, interests, and degree of impairment of people with dementia in nursing homes [38].

However, the findings show that the level of activity in nursing homes for people with dementia remains low [31]. Therefore, it is essential to offer meaningful activities and to increase the level of activity, for which professionals need knowledge, skills, and tools [39].

In summary, the design and delivery of individualized activities and occupations seem to be beneficial for older adults with dementia, as they facilitate the improvement of behavioral symptoms, anxiety and depression, cognitive functioning, social relationships, and QoL [21].

Second, intervention programs focusing on physical [22,23], cognitive, and sensory activities [24,25,26,27] have therapeutic effects on the physical function, mood, cognitive level, and QoL of residents.

Person-centered physical activity programs that provide physical activities tailored to each individual show moderate strength of evidence in improving the physical function, functional mobility, reduction of falls, ADLs, and QoL in older adults with dementia. In addition, they increase treatment adherence [22,23].

It should also be noted that multisensory stimulation activities complemented with individualized cognitive activities [24], and those based on reminiscence or recall [26,27], show moderate strength of evidence in improving the cognitive function, behavior, mood (depressive symptoms), and QoL of residents. However, music therapy and individualized listening to music show no significant effects on the behavioral and psychological symptoms of dementia [25].

These findings are supported by previous research which identified improvements in mental state and physical functioning associated with increased personal autonomy in older adults with dementia living in nursing homes [40,41].

In short, the design of physical, cognitive, and sensory activity programs seems to be effective in promoting the improved physical, cognitive, and emotional functioning and QoL of older adults with dementia living in nursing homes [22,27].

Third, intervention programs based on performance areas such as ADLs, IADLs, health management activities, work-related activities, and recreational and leisure activities [28,29,30] show therapeutic effects on the physical functioning, and behavioral and psychological symptoms of dementia and the QoL of the residents.

Personally tailored activities programs based on the performance of IADLs, work-related activities, and recreational and leisure activities show a high strength of evidence for the improvement of challenging behavior (restlessness, agitation, and aggression) of older adults with dementia [30].

In addition, programs for the improvement of ADLs performance and health management [28], as well as cooking activities in a group format [29], indicate moderate strength of evidence in improving the physical performance, behavior, executive function, and QoL of the residents.

These results are in line with those of Korczak et al. [42], which support the value of performance area-based activities, taking into account the individual’s functional ability to perform the activity and the degree of dementia, for the improvement of the behavior, functional independence, and QoL.

In short, the design and delivery of performance area-based activities seem to be beneficial for older adults with dementia, as they facilitate the improvement of the behavioral symptoms, physical function, functional independence, and QoL [28].

Finally, intervention programs aimed at modifying the physical and social environment and staff training [9,31,32] positively affect the comfort of the residents and thus their QoL.

The specialized training of staff and environmental modification programs show a high strength of evidence for the overall improvement of the QoL in older adults with dementia by improving functional independence and increasing the individual’s control over their immediate environment [9].

Moreover, the findings indicate with moderate strength of evidence [31,32] that such programs can significantly improve resident comfort.

In short, strategies aimed at improving the QoL in people over 65 years old with dementia should follow a two-fold approach. On the one hand, personalized programs that include ADLs, IADLs, recreational and leisure activities, and reminiscence activities, all of which with a strong social component, are required. On the other hand, adapting the residential environment is essential, with particular attention to the specialized training of the nursing home staff [9,32,43].

### 4.1. Implications for Practice, Policy, and Future Research

− The ability to choose meaningful activities and occupations in which the level of challenge is tailored to the type and stage of dementia and the functional capacity of the older adult with dementia to perform them are key elements in the design of intervention programs for the improvement of the QoL.
− It is essential to increase the level of activity of the residents. Therefore, modifications to residential environments are necessary, including a wider range and variety of activities, organizational changes that favor greater choice for older adults, and the provision of specialized training for healthcare professionals working in nursing homes.
− OT professionals could encourage older adults with dementia to participate in physical, cognitive, sensory, social, and performance area-based activities tailored to their needs, interests, and degree of impairment in order to enhance their well-being and QoL.
− Interventions focused on ADLs, IADLs, reminiscence activities, and recreational and leisure activities from a person-centered approach could improve the physical and cognitive functioning, behavioral and psychological symptoms of dementia, and QoL of residents.
− Future research should focus on such interventions, as well as on the formulation of new policies that consider such an approach.

### 4.2. Limitations

First, this review was limited by the heterogeneity of studies focused on improving the QoL of older adults with dementia living in nursing homes, in terms of the type, frequency, and duration of OT interventions; QoL measurements; and outcomes. Therefore, the impact of OT interventions on the QoL of this population cannot be fully ascertained. Second, articles indexed in other literature databases were excluded, which might have left out a significant number of related studies. Finally, only articles published in serialized journals were included, so unpublished articles or searches in the gray literature were not taken into account, which may be a valuable source for materials dealing with the specific review question.

## 5. Conclusions

OT intervention programs based on participation in recreational and free-time activities, reminiscence activities, performance-based activities and the physical and social environment, and specialized staff training, on a frequent and regular basis, and which take into account the interests and abilities of the residents could improve physical and cognitive functioning, behavioral and psychological symptoms of dementia, and the perceived QoL of older adults with dementia living in nursing homes. Therefore, we consider that the current findings can be used as a basis for the design of future intervention programs for the improvement of the QoL of older adults with dementia, as well as to inform care practices and service provision in nursing homes. However, due to the aforementioned limitations of this systematic review, the results should be viewed with caution, and improved studies are required. For future research, it would be necessary to unify the intervention programs in terms of frequency, duration, methodology, and the instruments used to measure QoL.

## Figures and Tables

**Table 1:** Characteristics of studies based on “Meaningful activities/occupations” intervention programs.

Author (Year)	Level of Evidence Study Design Risk of Bias	Participants Inclusion Criteria Study Setting	Intervention and Control Groups	Outcome Measures	Results
Travers et al. (2016) [18]	Level 1A Systematic review *Risk of bias* Low	*Participants* *N* = 3203 (*M* age, NR; % female, NR). *Inclusion criteria* Older adults over 65 years with dementia. *Study setting* Nursing homes	*Intervention: Promotion of meaningful activities* Recreational activities, music therapy, reminiscence therapy, sensory stimulation, animal-assisted therapy, and social participation. *Control group* Not applicable	Formal literature search in databases	*Significant findings* The promotion of meaningful activities successfully reduced agitation, passivity, and depression; increased pleasure and interest; and improved the QoL of older adults with dementia. *Not significant findings* None
Mansbach et al. (2017) [19]	Level 1B RCT *Risk of bias* Low	*Participants* *N* = 94 (*M* age, 82.9 years; 73.4% female). *Inclusion criteria* Older adults over 65 years with dementia. *Study setting* Nursing homes (USA)	*Intervention:* “*MemPics*” *program* (*n* = 48) Two weekly 30 min group sessions for 2 weeks consisting of verbal and cognitive stimulation activities. *Control group: Recreational activities* (*n* = 46) Two weekly 30 min group sessions for 2 weeks consisting of recreational activities (trivia questions, historical and current events, word games).	*QoL* MemPics Facility Staff Survey *Meaningful activities* EMAS *Cognitive functioning* BCAT-SF	*Significant findings* Significant differences were observed between groups with regard to participation in meaningful activities (EMAS) (F (1.92) = 4.72, *p* < 0.05). *Not significant findings* None
Livingston et al. (2019) [20]	Level 1B RCT *Risk of bias* Low	*Participants* *N* = 404 (*M* age, 86 years; 71.5% female). *Inclusion criteria* Older adults over 65 years with dementia. *Study setting* Nursing homes (UK)	*Intervention:* “*MARQUE*” *program* (*n* = 189) Three weekly 60 min group sessions for 2 weeks based on agitation management and improvement of QoL. *Control group: Usual care* (*n* = 215) Three weekly 60 min group sessions for 2 weeks based on usual care.	*QoL* - DEMQOL - EQ-5D-5L *Behavior* CMAI	*Significant findings* No significant differences were found between groups for agitation (difference −0.40, 95% CI: −3.89 to 3.09; *p* = 0.82) and QoL (difference = 0.09, 95% CI: −3.87 to 4.05; *p* = 0.96). *Not significant findings* None
Sultan Ibrahim et al. (2021) [21]	Level 1B RCT *Risk of bias* Low	*Participants* *N* = 32 (*M* age, 75.9 years; 25% female). *Inclusion criteria* Older adults over 65 years with dementia. *Study setting* Nursing homes (Malaysia)	*Intervention:* “*Occupation-based intervention*” *program* (*n* = 16) Two weekly 60 min group sessions for 7 weeks consisting of meaningful occupational and cognitive activities. *Control group: Usual care* (*n* = 16) Two weekly 60 min group sessions for 7 weeks of conventional OT.	*QoL* WHOQOL-BREF *Cognitive functioning* LOTCA-G *Social relations* FS	*Significant findings* The intervention group showed statistically significant improvements in QoL, cognitive function, and social relations (*p* = 0.02). *Not significant findings* None

*Note*. BCAT-SF = Brief Cognitive Assessment Tool-Short Form; CMAI = Cohen-Mansfield Agitation Inventory; DEMQOL = Dementia Quality of Life; EMAS = Engagement in Meaningful Activities Survey; EQ-5D-5L = EuroQol-5D-5L; FS = Friendship Scale; LOTCA-G = Lowenstein Occupational Therapy Cognitive Assessment-Geriatric; NR = Not Reported; WHOQOL-BREF = Brief Version of World Health Organization.

**Table 2:** Characteristics of studies based on “Physical, cognitive and sensory functioning” intervention programs.

Author (Year)	Level of Evidence Study Design Risk of Bias	Participants Inclusion Criteria Study Setting	Intervention and Control Groups	Outcome Measures	Results
Galik et al. (2014) [22]	Level 1B RCT *Risk of bias* Low	*Participants* *N* = 103 (*M* age, 83.7 years; 77% female). *Inclusion criteria* Older adults over 65 years with dementia. *Study setting* Nursing homes (USA)	*Intervention: Function-focused care intervention for older adults with dementia (FFC-CI)* (*n* = 51) Five weekly 45 min group sessions for 24 weeks consisting of an assessment of the physical environment, education program for nursing home staff, development of function-focused care goals, and mentoring and motivation. *Control group: Function-focused care based on education (FFC-ED)* (*n* = 52) Five weekly 30 min group sessions for 24 weeks consisting exclusively of a training program for nursing home residents and staff.	*Physical function* - Tinetti Scale - BI *Physical activity* ActiGraph *Behavior* CMAI *Depression* CSDD *Apathy* Apathy Evaluation Scale	*Significant findings* The intervention group showed statistically significant improvements in physical function (from 45.56 to 55.20, *p* = 0.01), and in the amount and intensity of physical activity (from 115.96 min to 126.05 min, *p* = 0.01, and from 20,309 to 86,288, *p* = 0.01), and had fewer falls (28% vs. 50% in the control group). *Not significant findings* No significant differences were found between groups with regard to psychosocial outcomes, agitation, depression, or apathy.
Chu et al. (2020) [23]	Level 2B Quasi-experimental *Risk of bias* Moderate	*Participants* *N* = 26 (*M* age, 86.8 years; 80.8% female). *Inclusion criteria* Older adults over 65 years with dementia. *Study setting* Nursing homes (Canada)	*Intervention:* “*Multifaceted Walking Intervention*” *(MWI) program* (*n* = 15) Four weekly individual sessions for 16 weeks consisting of low-intensity physical activity and an individualized care plan. *Control group: Usual care* (*n* = 11) Four weekly individual sessions for 8 weeks consisting of usual care.	*QoL* ADRQOL *Physical function* - TUG - 2MWT - Gait Speed *ADLs* FIM	*Significant findings* The intervention group showed significant improvements in QoL (*p* = 0.057), functional mobility (measured with the TUG; improvement of 32.14%, *p* = 0.000; assessed with the 2MWT, improvement from 53.60 to 81.07, *p* = 0.000), gait speed (evaluated with the Gait Speed test; improvement of 55.11%, *p* = 0.000), and ADLs (assessed with the FIM scale; improvement of 25%, *p* = 0.000). *Not significant findings* None
Maseda et al. (2014) [24]	Level 1B RCT *Risk of bias* Low	*Participants* *N* = 30 (*M* age, 87.3 years; 90% female). *Inclusion criteria* Older adults over 65 years with dementia. *Study setting* Nursing homes (Spain)	*Intervention 1: Multisensory stimulation environment (MSSE)* (*n* = 10) Two weekly 30 min individual sessions for 16 weeks held in a “Snoezelen” room with elements to stimulate the senses. *Intervention 2: Individual activities* (*n* = 10) Two weekly 30 min individual sessions for 16 weeks in which intellectual and/or physical demands were placed on the individual through cognitive and recreational activities. *Control group: Usual care* (*n* = 10) Two weekly 30 min group sessions for 16 weeks of conventional OT (e.g., cognitive stimulation, training in ADLs).	*Behavior* - CMAI - NPINH *Cognitive functioning* - MMSE - CSDD *Depression* GDS *ADLs* BI	*Significant findings* The intervention group showed significant improvements in behavior (F (2.36) = 4.513, *p* = 0.18), cognitive level (F (1.12) = 5.457, *p* = 0.038), and ADLs (from 33 to 47 points). *Not significant findings* No improvement in mood was observed.
Raglio et al. (2015) [25]	Level 1B RCT *Risk of bias* Low	*Participants* *N* = 120 (*M* age, 81.7 years; 78.3% female). *Inclusion criteria* Older adults over 65 years with dementia. *Study setting* Nursing homes (Italy)	*Intervention 1: Standard care (SC) + music therapy (MT)* (*n* = 40) Two weekly 30 min individual sessions for 10 weeks consisting of physical, educational, and occupational activities + music therapy. *Intervention 2: Standard care (SC) + individualized listening to music (LtM)* (*n* = 40) Two weekly 30 min individual sessions for 10 weeks consisting of physical, educational, and occupational activities + individualized listening to music. *Control group: Standard care (SC)* (*n* = 40) Two weekly 30 min individual sessions for 10 weeks consisting exclusively of physical, educational, and occupational activities (no musical exposure).	*QoL* CBS-QoL *Depression* CSDD *Behavior* NPI	*Significant findings* All groups had statistically significant improvements in depression (measured with the CSDD; *p* = 0.001), behavior (assessed with the NPI; *p* = ≤0.001), and QoL (assessed with the CBS-QoL; *p* = 0.01). *Not significant findings* None
Lök et al. (2019) [26]	Level 1B RCT *Risk of bias* Low	*Participants* *N* = 60 (*M* age, NR; 56.7% female). *Inclusion criteria* Older adults over 65 years with dementia. *Study setting* Nursing homes (Turkey)	*Intervention: Reminiscence therapy* (*n* = 30) One weekly 60 min group session for 8 weeks, consisting of recalling memories of relevant experiences, positive experiences, and achievements from the past. *Control group: No intervention* (*n* = 30) No intervention was provided.	*QoL* QOL-AD *Depression* CSDD *Cognitive functioning* SMMSE	*Significant findings* The intervention group showed statistically significant improvements in QoL, depression, and mental state (*p* < 0.05). *Not significant findings* None
Kim (2020) [27]	Level 1B RCT *Risk of bias* Low	*Participants* *N* = 35 (*M* age, 79.2 years; 74.3% female). *Inclusion criteria* Older adults over 65 years with dementia. *Study setting* Nursing homes (South Korea)	*Intervention: OT program based on recall* (*n* = 18) Five weekly 60 min sessions for 5 weeks. Nine programs were carried out for each activity (physical activity, music, art, horticulture, and IADLs). *Control group: Usual care* (*n* = 17) Five weekly 60 min sessions for 5 weeks consisting of physical activity, recreational, educational and occupational activities, and music therapy.	*QoL* GQOL-D *ADLs* FIM *Cognitive functioning* - K-MMSE - SMCQ *Depression* SGDS-K	*Significant findings* The intervention group showed statistically significant improvements in subjective memory impairment (from 5.83 to 4.16, *p* < 0.05), cognitive function (from 18.70 to 19.56, *p* < 0.05), depression (from 6.55 to 4.1, *p* < 0.05), and QoL (from 30.11 to 33.5, *p* < 0.05). *Not significant findings* None

*Note*. ADRQOL = Alzheimer’s Disease-Related Quality of Life Scale; BI = Barthel Index; CBS-QoL = Cornell-Brown Scale for Quality of Life in Dementia; CMAI = Cohen-Mansfield Agitation Inventory; CSDD = Cornell Scale for Depression in Dementia; FIM = Functional Independent Measure; GDS = Geriatric Depression Scale; GQOL-D = Geriatric Quality of Life-Dementia; K-MMSE = Korean-Mini-Mental State Examination; MMSE = Mini-Mental State Examination; 2MWT = 2-Minute Walk Test; NPI = Neuropsychiatric Inventory; NPI-NH = Neuropsychiatric Inventory–Nursing Home; QOL-AD = Quality of Life in Alzheimer’s Disease-Patient and Caregiver Report; SGDS-K = Short-Form Geriatric Depression Scale-K; SMCQ = Subjective Memory Complaints Questionnaire; SMMSE = Standardized Mini-Mental State Examination; TUG = Timed Up and Go Test.

**Table 3:** Characteristics of studies based on “Performance areas” intervention programs.

Author (Year)	Level of Evidence Study Design Risk of Bias	Participants Inclusion Criteria Study Setting	Intervention and Control Groups	Outcome Measures	Results
Kumar et al. (2014) [28]	Level 1B RCT *Risk of bias* Low	*Participants* *N* = 77 (*M* age, 69.4 years; 19.5% female). *Inclusion criteria* Older adults over 65 years with dementia. *Study setting* Nursing homes (India)	*Intervention: OT intervention + standard medical treatment* (*n* = 36) Two weekly 70 min individual sessions for 5 weeks consisting of relaxation exercises, physical activity, cognitive and recreational activities, ADLs and IADLs + pharmacological treatment. *Control group: Standard medical treatment* (*n* = 41) Individual pharmacological treatment for 5 weeks.	*QoL* WHOQOL-BREF Standard OT assessment	*Significant findings* The intervention group had significant improvements in the overall QoL domain (from 66.78 to 71.36, *p* < 0.001), physical domain (from 37.30 to 45.43, *p* < 0.001), environmental domain (from 37.76 to 38.62, *p* = 0.006), and psychological domain (from 45.13 to 51.50, *p* < 0.001). *Not significant findings* The intervention group showed no significant improvements in the social relations domain.
Murai & Yamaguchi (2017) [29]	Level 1B RCT *Risk of bias* Low	*Participants* *N* = 36 (*M* age, 85.4 years; 80.6% female). *Inclusion criteria* Older adults over 65 years with dementia. *Study setting* Nursing homes (Japan)	*Intervention: Cooking program* (*n* = 16) One weekly 90 min group session for 12 weeks consisting of cooking a Japanese-style menu. *Control group: Recreational activities* (*n* = 16) One weekly 90 min group session for 12 weeks consisting of recreational activities (volleyball, radio, gymnastics, choir).	*QoL* PGC *Behavior* DBD *ADLs* BI *Depression* GDS *Executive function* YKSST	*Significant findings* Significant differences were found between groups for executive function (measured with the YKSST) (F (1.27) = 4.305, *p* = 0.048) and behavior (assessed with the DBD scale) (F (1.29) = 13.298, *p* = 0.001). *Not significant findings* No significant differences were observed between groups regarding QoL, depression, and ADLs.
Möhler et al. (2018) [30]	Level 1A Systematic review *Risk of bias* Low	*Participants* *N* = 957 (*M* age, 83 years; % female, NR). *Inclusion criteria* Older adults over 65 years with dementia. *Study setting* Nursing homes	*Intervention: Promotion of personally tailored activities* IADLs, and recreational, artistic, and work-related activities. *Control group* Not applicable	Formal literature search in databases	*Significant findings* Offering personalized activities could improve the challenging behavior of older adults with dementia (standardized mean difference = −0.21, 95% CI: −0.49 to 0.08; I^2^ = 50%). *Not significant findings* None

*Note*. BI = Barthel Index; DBD = Dementia Behavior Disturbance; GDS = Geriatric Depression Scale; PGC = Philadelphia Geriatric Center Morale Scale; WHOQOL-BREF = Brief Version of World Health Organization; YKSST = Yamaguchi Kanji-Symbol Substitution Test.

**Table 4:** Characteristics of studies based on “Physical and social environment and staff training” intervention programs.

Author (Year)	Level of Evidence Study Design Risk of Bias	Participants Inclusion Criteria Study Setting	Intervention and Control Groups	Outcome Measures	Results
Wenborn et al. (2013) [31]	Level 1B RCT *Risk of bias* Low	*Participants* *N* = 159 (*M* age, 84.2 years; 67.2% female). *Inclusion criteria* Older adults over 65 years with dementia. *Study setting* Nursing homes (UK)	*Intervention: OT program* (*n* = 79) One 120 min group session every 3 weeks for 16 weeks consisting of an assessment of the physical environment of the nursing home, a training program for the nursing home staff, and the implementation of new personally meaningful activities. *Control group: Usual care* (*n* = 80) One 120 min group session every 3 weeks for 16 weeks, with no specific focus on training or new activities.	*QoL* - QOL-AD Patient - QOL-AD Caregiver *Dependence* CAPE-BRS *Challenging behavior* CBS *Depression* CSDD *Anxiety* RAID	*Significant findings* The staff-rated QoL (measured with the QOL-AD Caregiver) was slightly lower in the intervention group (mean difference of staff ratings = −1.91, 95% CI: −3.39 to −0.43, *p* = 0.01). *Not significant findings* No significant differences were found between groups for self-rated QoL (assessed with the QOL-AD Patient), dependence, challenging behavior, depression, and anxiety.
Ojagbemi & Owolabi, (2017) [9]	Level 1A Systematic review *Risk of bias* Low	*Participants* *N* = 1002 (*M* age, 78.6 years; 53% female). *Inclusion criteria* Older adults over 65 years with dementia. *Study setting* Nursing homes	*Intervention: OT intervention* Free time, sensorimotor and compensatory activities, IADLs, cognitive and relaxation exercises, staff training, and environmental modification. *Control group* Not applicable	Formal literature search in databases	*Significant findings* The exclusive use of OT interventions resulted in a slight overall improvement in the QoL of older adults with dementia. *Not significant findings* None
Froggatt et al. (2020) [32]	Level 1B RCT *Risk of bias* Low	*Participants* *N* = 32 (*M* age, 81.5 years; 47% female). *Inclusion criteria* Older adults over 65 years with dementia. *Study setting* Nursing homes (UK)	*Intervention:* “*Namaste Care*” *program* (*n* = 18) Seven weekly 120 min group sessions for 24 weeks, consisting of creative activities, multisensory stimulation, social participation, and a training program for the nursing home staff. *Control group: Usual care* (*n* = 14) Seven weekly 120 min group sessions for 24 weeks consisting of usual care.	*QoL* - QUALID - CAD-EOLD	*Significant findings* The intervention group showed significant improvements in comfort (measured with the CAD-EOLD scale) (from 34.8% to 37.6%). *Not significant findings* No significant differences were found between groups for QoL (assessed with the QUALID scale).

*Note*. CAD-EOLD = Comfort Assessment in Dying-End of Life Care in Dementia; CAPE-BRS = Clifton Assessment Procedures for the Elderly-Behavior Rating Scale; CBS = Challenging Behavior Scale; CSDD = Cornell Scale for Depression in Dementia; QOL-AD = Quality of Life in Alzheimer’s Disease-Patient and Caregiver Report; QUALID = Quality of Life in Late Stage Dementia; RAID = Rating Anxiety in Dementia.

**Table 5:** Risk-of-Bias Table for Systematic Reviews (AMSTAR 2).

Citation	1	2	3	4	5	6	7	8	9	10	11	12	13
Travers et al. [18]	+	+	+	+	+	**+**	+	+	**+**	+	+	**+**	L
Ojagbemi & Owolabi (2017) [9]	+	+	+	+	+	+	+	+	+	+	+	+	L
Möhler et al. [30]	+	+	+	+	+	**+**	+	**+**	**+**	**+**	+	**+**	L

*Note.* 1: All components of PICO addressed; 2: “a priori design” included? 3: Explanation of the selection of the study designs for inclusion in the review? 4: Comprehensive literature search performed? 5: Authors perform study selection and data extraction in duplicate? 6: List of excluded studies provided? 7: Authors describe the included studies in adequate detail? 8: Quality of studies (risk of bias) assessed and documented? 9: Authors report on the sources of funding for the studies included in the review? 10: Authors account for risk of bias in primary studies when interpreting/discussing the results of the review? 11: Satisfactory explanation for, and discussion of, any heterogeneity observed in the results of the review? 12: Authors report any potential sources of conflict of interest, including any funding they received for conducting the review? 13: Overall risk of bias assessment (low, moderate, high risk). *Citation.* Table format adapted from Shea et al. [34].

**Table 6:** Risk-of-Bias Table for Randomized Controlled Trial (RCT) and Non-RCT.

	Selection Bias (Risk of Bias Arising from Randomization Process)			Performance Bias (Effect of Assignment to Intervention)		Detection Bias		Attrition Bias	Reporting Bias	Overall Risk of Bias Assessment
Citation	1	2	3	4	5	6	7	8	9	10
Wenborn et al. [31]	+	+	+	?	?	+	+	+	+	L
Galik et al. [22]	+	+	+	?	?	+	+	+	+	L
Kumar et al. [28]	+	+	+	+	+	+	**-**	+	+	L
Maseda et al. [24]	+	+	+	+	+	+	+	+	+	L
Raglio et al. [25]	+	+	+	+	?	+	+	+	+	L
Murai & Yamaguchi [29]	+	+	+	+	?	+	+	+	+	L
Mansbach et al. [19]	+	+	+	+	+	+	+	+	+	L
Lök et al. [26]	+	+	+	?	**-**	+	+	+	+	L
Livingston et al. [20]	+	+	+	+	**-**	+	+	+	+	L
Chu et al. [23]	**-**	**-**	+	**-**	**-**	**-**	+	+	+	M
Froggatt et al. [32]	+	+	+	**-**	**-**	+	**-**	+	+	L
Kim et al. [27]	+	+	+	+	?	+	**+**	+	+	L
Sultan Ibrahim et al. [21]	+	+	+	+	+	+	+	+	+	L

*Note.* 1: Random sequence generation; 2: Allocation concealment (until participants enrolled and assigned); 3: Baseline differences between intervention groups (suggest problem with randomization?); 4: Blinding of participants during the trial; 5: Blinding of study personnel during the trial; 6: Blinding of outcome assessment: self-reported outcomes; 7: Blinding of outcome assessment: objective outcomes (assessors aware of intervention received?); 8: Incomplete outcome data (data for all or nearly all participants); 9: Selective reporting (results being reported selected on the basis of the results?); 10: Overall risk of bias assessment (low, moderate, high risk). *Citation*. Table format adapted from Higgins et al. [33].

## Data Availability

Not applicable.
